# Cholinesterase inhibitor rivastigmine enhances nerve growth factor-induced neurite outgrowth in PC12 cells via sigma-1 and sigma-2 receptors

**DOI:** 10.1371/journal.pone.0209250

**Published:** 2018-12-17

**Authors:** Kazuki Terada, Keisuke Migita, Yukari Matsushima, Yumi Sugimoto, Chiaki Kamei, Taichi Matsumoto, Masayoshi Mori, Kazuhisa Matsunaga, Jiro Takata, Yoshiharu Karube

**Affiliations:** 1 Department of Pharmaceutical Sciences, Fukuoka University, Fukuoka, Japan; 2 Faculty of Pharmaceutical Sciences, Yasuda Women’s University, Hiroshima, Japan; 3 Faculty of Pharmaceutical Sciences, Himeji Dokkyo University, Hyogo, Japan; Chiba Daigaku, JAPAN

## Abstract

Rivastigmine (Riv) is a potent and selective cholinesterase (acetylcholinesterase, AChE and butyrylcholinesterase, BuChE) inhibitor developed for the treatment of Alzheimer’s disease (AD). To elucidate whether Riv causes neuronal differentiation, we examined its effect on nerve growth factor (NGF)-induced neurite outgrowth in PC12 cells. At concentrations of 0–100 μM, Riv was non-toxic in PC12 cells. Riv caused dose-dependent (10–100 μM) enhancement of NGF-induced neurite outgrowth, which was completely inhibited by the TrkA antagonist GW-441756. By contrast, Riv-mediated enhancement of neurite outgrowth was not blocked by the acetylcholine receptor antagonists, scopolamine and hexamethonium. However, the sigma-1 receptor (Sig-1R) antagonist NE-100 and sigma-2 receptor (Sig-2R) antagonist SM-21 each blocked about half of the Riv-mediated enhancement of NGF-induced neurite outgrowth. Interestingly, the simultaneous application of NE-100 and SM-21 completely blocked the enhancement of NGF-induced neurite outgrowth by Riv. These findings suggest that both Sig-1R and Sig-2R play important roles in NGF-induced neurite outgrowth through TrkA and that Riv may contribute to neuronal repair via Sig-1R and Sig-2R in AD therapy.

## Introduction

Alzheimer’s disease (AD) is a progressive neurodegenerative dementia characterized by impaired memory and cognition [1]. The main pathological findings of AD are brain atrophy, amyloid deposition, and neurofibrillary degeneration [2]. Cholinergic neurons of the central nervous system are known to undergo selective and severe degeneration in AD [3]. One possible therapeutic treatment for AD is to compensate for the decrease in cholinergic system activity in the basal forebrain [4]. Therefore, acetylcholinesterase (AChE) inhibitors such as donepezil (DNP), rivastigmine (Riv), and tacrine (Tac) have been used as AD therapeutic agents [5]. Clinically, randomized controlled trials comparing the effects of DNP and Riv have reported that both drugs function similarly in terms of cognition and behavior [6]. Recently, it has been shown that Riv has a beneficial effect on neuropsychiatric features and depression [6,7]. Non-clinically, it was reported that AChE inhibitors protect against glutamate-induced neurotoxicity via the neuronal nicotinic acetylcholine receptor (AchR) [8,9]. It has also been discovered that DNP induces the differentiation of neurons via the sigma-1 receptors (Sig-1R) [10]. This effect does not depend upon the AChE inhibitory action. The pathway through sigma receptors (Sig-R) may strengthen the pharmacological effect of AD therapeutic drugs in the human brain [11].

Sig-R were initially proposed to be a subtype of opioid receptor and were demonstrated in subsequent studies to be unique proteins highly conserved across species, cell types, and organelles [12–14]. In the brain, Sig-R are distributed in the limbic and endocrine domains that are involved in the pathophysiology of depression and AD, such as the hippocampus, frontal cortex, hypothalamus, and olfactory bulb [15–17]. Two subtypes of Sig-R have been identified, termed sigma-1 and sigma-2 receptors (Sig-1R and Sig-2R, respectively) [12]. These subtypes are distinguished by their function, molecular size, and pharmacological profile [18,19]. Studies of Sig-R have been biased toward the Sig-1R subtype [20–22]. Therefore, the biological role and contribution of Sig-2R are thus far virtually unknown [23]. However, some data have recently shown that Sig-2R is a promising therapeutic target for neurocognitive disorders, including AD [24–26]. More recently, Sig-2R was cloned and identified as transmembrane protein 97 (TMEM97) [27].

PC12 is a cell line derived from a pheochromocytoma of the rat adrenal medulla, and it has been widely used as a model system for nerve growth factor (NGF)-induced neuronal differentiation [28–30]. Sig-1R and Sig-2R are known to be expressed in PC12 cells [31,32]. It has been reported that Sig-1R agonists such as DNP, fluvoxamine, and imipramine promote NGF-induced neurite outgrowth in PC12 cells and that elongation is inhibited by the selective Sig-1R antagonist NE-100 [10,33]. However, it is not yet known whether Riv participates in neurite outgrowth through Sig-R.

In this study, we examined whether the effect of Riv on NGF-induced neurite outgrowth in PC12 cells is associated with Sig-1R and Sig-2R. Our findings demonstrate that Riv enhances NGF-induced neurite outgrowth via both Sig-1R and Sig-2R without promoting AChE inhibitory action in PC12 cells.

## Materials and methods

### Materials

Riv (rivastigmine tartrate) was purchased from Sigma-Aldrich (St. Louis, MO, USA). Murine NGF 2.5S (NGF derived from mouse submaxillary glands) was obtained from Alomone Labs (Jerusalem, Israel). Scopolamine (scopolamine hydrobromide trihydrate, Scop) and hexamethonium (hexamethonium bromide, HEX) were purchased from Tokyo Chemical Industry Co., Ltd (Tokyo, Japan). The Sig-1R antagonist NE-100 (NE-100 hydrochloride) and the AChE inhibitor physostigmine (Phy) were obtained from Santa Cruz Biotechnology (Santa Cruz, CA, USA). The Sig-2R antagonist SM-21 (SM21 maleate) and TrkA receptor antagonist GW-441756 (GW-441756 hydrochloride) were purchased from Abcam (Cambridge, MA, USA).

### Cell culture

PC12 cells obtained from the Riken Cell Bank (Ibaraki, Japan) were maintained in Dulbecco’s modified Eagle’s medium (DMEM)/F-12 supplemented with 10% (v/v) fetal bovine serum (FBS; Gibco, Life Technologies, Franklin Lakes, NJ, USA) and 1% (v/v) penicillin–streptomycin. Cells were kept in an incubator at 37°C in an atmosphere of 5% CO_2_/95% air.

### Cell viability assay

PC12 cells were seeded into 96-well plates (Corning Inc., Corning, NY, USA) at a density of 1.0 × 10^4^ cells/well for 24 h. After incubation of PC12 cells with various concentrations of Riv (0.1–1000 μM) for an additional 24 h, cell viability was assessed using the cell proliferation reagent WST-1 (Roche Applied Science, Mannheim, Germany) according to the manufacturer’s instructions. Briefly, the culture medium was removed from wells after treatment, and 100 μL of medium containing 10 μL WST-1 was added to each well. Absorbance at 450 nm was determined after 4 h.

### Measurement of neurite outgrowth

The neurite outgrowth of PC12 cells was measured following the method described by Terada et al. [30,34]. PC12 cells were seeded into type I collagen-coated 60-mm tissue culture dishes (Iwaki, Tokyo, Japan) at a density of 1.5 × 10^5^ cells/dish in DMEM/F-12 containing 10% FBS for 24 h. After incubation, PC12 cells were stimulated with differentiation medium (DMEM/F-12 containing 5% FBS plus 50 ng/mL NGF with or without either Riv at 0.1–100 μM, Phy at 100 μM, or Sig-1R agonist PRE-084 and Sig-2R agonist PB28 at 10 μM). Cells were also treated with various concentrations of NGF (5–250 ng/mL) in the absence or presence of Riv (100 μM). Neurite extension length was measured 24 h after the administration of treatment regimens. Cells were incubated for an additional 24 h and photographed using a digital camera (Digital Sight DS-L2 system; Nikon, Tokyo, Japan) under a phase-contrast microscope (ECLIPSE TS100; Nikon).

Images of five randomly selected fields per dish were obtained, with a mean number of 15–20 PC12 cells per field. The total length of the neurites extending from all cells in each of the five fields was automatically measured using ImageJ software (National Institutes of Health, Bethesda, MD, USA). The average neurite length per field was obtained by dividing the total neurite length by the number of cells per field. Finally, the results from all fields examined for each condition were averaged (n = 6) to yield an average neurite length per condition. Values for maximal response (E_max_), indicating maximum neurite outgrowth length, and the concentration of NGF resulting in the half E_max_ (EC_50_) were determined using GraphPad Prism 6.0 (GraphPad Software, San Diego, CA, USA) according to the E_max_ model for the concentration–response curve equation: E = E_max_ × ([NGF] / (EC_50_ + [NGF])), where E is neurite length. Unless otherwise specified, data are presented as mean ± standard deviation (SD), 95% confidence interval (CI), or coefficient of determination (R^2^) of the mean value, as appropriate.

### Pharmacological inhibition of several receptors in PC12 cells

To evaluate the influence of the blockade of the TrkA receptor, muscarinic acetylcholine receptor, nicotinic acetylcholine receptor, Sig-1R, or Sig-2R on the NGF-mediated regulation of neurite outgrowth, PC12 cells were individually or simultaneously pretreated with a pharmacological antagonist of each receptor—GW-441756 (1 μM), Scop (100 μM), HEX (100 μM), NE-100 (10 μM), and SM-21 (10 μM), respectively—4 h prior to the addition of NGF (50 ng/mL) with or without Riv (100 μM). Cells were incubated with NGF for another 24 h, and neurite outgrowth was analyzed as described above.

### Western blotting analysis

Western blotting analyses were performed as previously described [30]. Protein samples containing 8 μg of total protein were separated via electrophoresis with 4–15% sodium dodecyl sulfate (SDS)-polyacrylamide gels, after which they were transferred onto polyvinylidene fluoride (PVDF) membranes (Bio-Rad, Hercules, CA, USA). After transfer, PVDF membranes were blocked with 5% bovine serum albumin (Wako Pure Chemical Industries, Osaka, Japan) in TBS containing 0.1% Tween-20 at room temperature for 1 h.

Immunoblotting was then performed using primary antibodies against phospho-Akt (p-Akt) (Ser473; 1:1000, Cell Signaling Technology, Danvers, MA, USA), Akt (1:1000, Cell Signaling Technology), phospho-ERK1(T202/Y204)/ERK2(T185/Y187) (p-ERK1/2) (1:2000, R&D Systems, Minneapolis, MN, USA), and ERK1/2 (1:2000, R&D Systems). Horseradish peroxidase-conjugated secondary antibody was used to detect immunoreactivity (Amersham Pharmacia Biotech, Piscataway, NJ, USA), which was visualized using enhanced chemiluminescence western blotting detection reagents (Amersham Pharmacia Biotech) and RX-U Fuji X-ray film (Fuji Film, Tokyo, Japan). Data were analyzed using ImageJ software.

### siRNA transfection

Small interfering RNA (siRNA) was purchased from Applied Biosystems (Stealth select siRNA; Foster City, CA, USA). The targeting sense sequence for rat *Sig-1R* in PC12 cells was 5′-CACCCUCUUCUAUACCCUUtt-3′, and that for rat *TMEM97* (Sig-2R) in PC12 cells was 5′-CUGUUGCGGUGGUACUCUAtt -3′. Stealth RNAi Negative Control Duplex (Applied Biosystems) was used as a negative control (scrambled siRNA) for the RNAi response. All were transfected using Lipofectamine RNAi MAX (Thermo Fisher Scientific, Inc., Waltham, MA, USA) in Opti-MEM (Thermo Fisher Scientific) according to the manufacturer’s instructions. Cells were used for experiments 48 h after transfection.

### Statistical analysis

Quantitative data are given as means ± SD. Statistical analysis of quantitative data was performed using analysis of variance (ANOVA) tests followed by Tukey’s post-hoc tests. In all cases, *P <* 0.05 was considered statistically significant.

## Results

### Influence of Riv on cell viability

**Fig 1:**
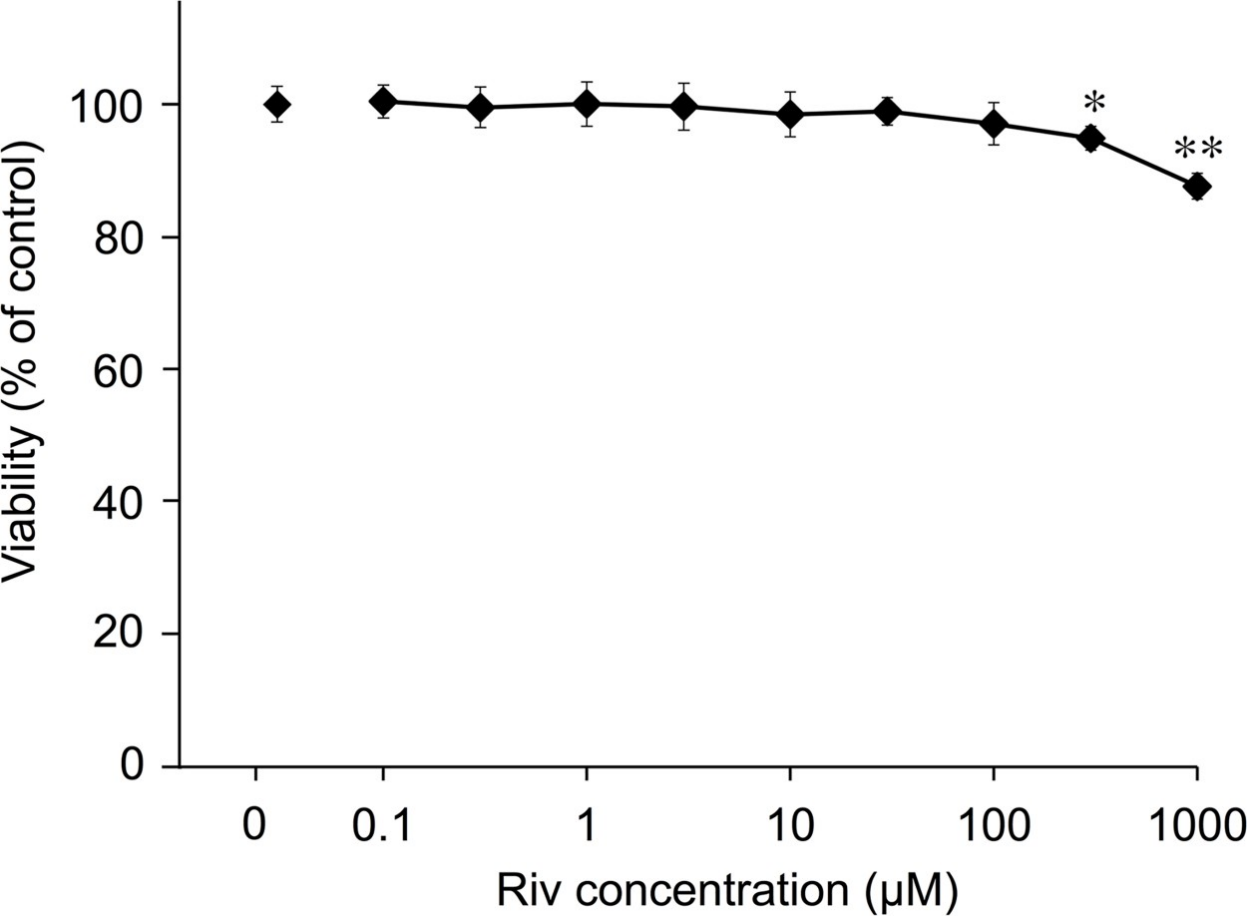
Influence of Riv on PC12 cell viability. PC12 cells were treated with Riv for 24 h. Cell viability was determined using a WST-1 assay, and the results are expressed as a percentage of the control value (0 μM). Experiments were repeated at least three times, and the values represent the mean of three experiments ± SD. *p < 0.05, **p < 0.01 versus control.

We investigated the effect of Riv on the viability of PC12 cells using a WST-1 assay. Riv did not affect cell proliferation at concentrations below 100 μM, but cytotoxicity was observed at 300 μM and above (Fig 1). Therefore, we used Riv in concentrations of 0.1–100 μM for subsequent experiments.

### Riv enhances NGF-induced neurite outgrowth in PC12 cells

**Fig 2:**
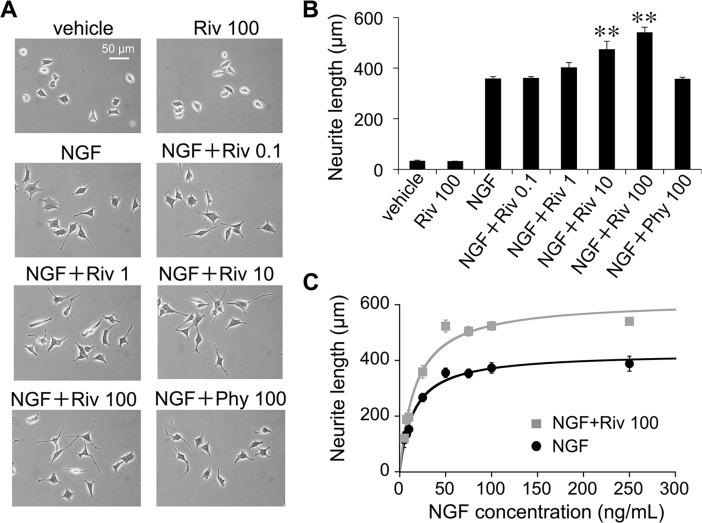
Riv enhances NGF-induced neurite outgrowth in PC12 cells. PC12 cells were treated for 24 h with NGF with or without Riv. (**A**) Culture images at a magnification of 400×. Scale bar: 50 μm. (**B**) Neurite length was determined as indicated in the Materials and Methods section. Values represent the mean ± SD for all PC12 cells contained within five randomly chosen fields for each condition. All experiments were repeated at least three times **p < 0.01 versus NGF. (**C**) Neurite lengths in the presence of various concentrations of NGF (0–250 ng/mL).

To investigate the effect of Riv on NGF-induced neurite outgrowth, PC12 cells were cultured for 24 h in the absence or presence of NGF with Riv (0.1–100 μM). Similar to the findings of previous studies [30], treatment with NGF alone significantly increased total neurite outgrowth, but little neurite outgrowth was observed in cells treated with vehicle or 100 μM Riv alone (Fig 2A and 2B). NGF-induced neurite outgrowth was significantly enhanced by treatment with 10 and 100 μM Riv. By contrast, treatment with the peripheral AChE inhibitor Phy did not enhance NGF-induced neurite outgrowth (Fig 2A and 2B).

Riv significantly enhanced NGF-induced elongation throughout the tested NGF concentration range (5–250 ng/mL, Fig 2C). E_max_ responses differed significantly following NGF and NGF+Riv treatment (427.3 ± 12.8 μm, 95% CI: 396.1–458.5; 616.1 ± 29.9 μm, 95% CI: 543.0–689.2, respectively); however, the concentrations of NGF corresponding to the EC_50_ values for these two conditions were not significantly different (15.1 ± 1.7 ng/mL, 95% CI: 11.0–19.2; 17.3 ± 3.0, 95% CI: 9.9–24.7 ng/mL, respectively). Curve fitting analysis of the observed plots using an E_max_ model provided comparatively good coefficients of determination for both the NGF and NGF+Riv groups (R^2^: 0.98528 and 0.96912, respectively). Thus, Riv increased the E_max_ for neurite outgrowth induced by NGF treatment, whereas no significant change was observed for the EC_50_ of NGF. These results suggest that the enhancing effects of Riv were not additive with those of NGF, but cooperative. That is, if Riv had NGF receptor agonistic activity, the E_max_ would remain unchanged.

### Riv potentiates NGF-induced phosphorylation of Akt and ERK1/2

**Fig 3:**
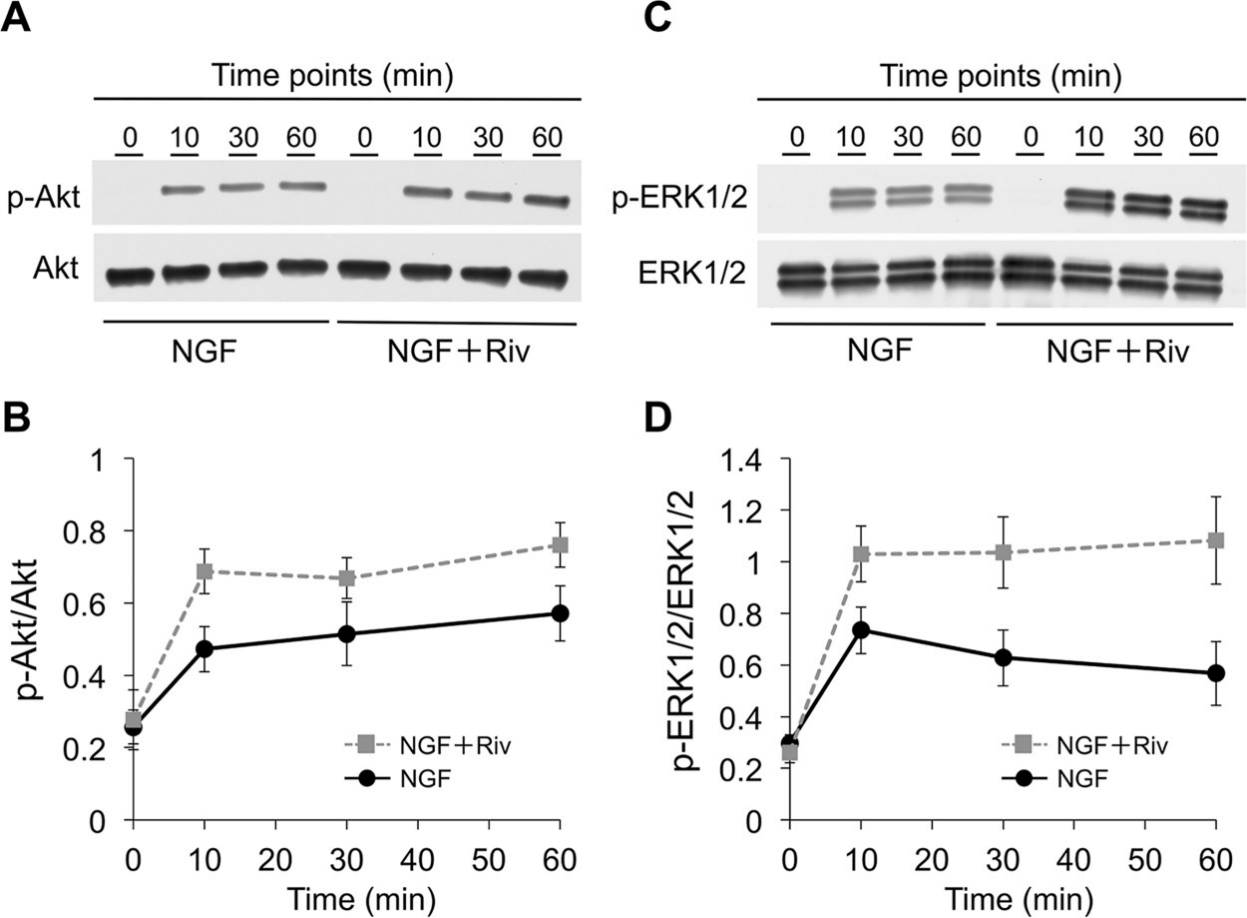
Influence of Riv on NGF-induced phosphorylation of Akt and ERK1/2 in PC12 cells. PC12 cells underwent various durations of treatment (0, 10, 30, or 60 min) with NGF in the presence or absence of Riv (100 μM) (**A**, **C**) Total protein lysates were collected and subjected to western blotting for detection of phosphorylation status of Akt and ERK1/2. (**B**, **D**) The intensity of the p-Akt polypeptide band was normalized to that of the total Akt band (p-Akt/Akt) (**B**), while the intensity of the p-ERK1/2 polypeptide band was normalized to that of the total ERK1/2 band (p-ERK1/2/ERK1/2) (**D**). Results are presented as the mean ± SD of three independent experiments.

It is well known that phosphorylation of Akt and ERK1/2 is associated with NGF-induced neurite outgrowth [35]. We therefore evaluated the time course of the phosphorylation of Akt and ERK1/2 in PC12 cells stimulated by NGF in the absence and presence of Riv using western blotting. Levels of p-Akt gradually increased 10 min after the addition of NGF in the absence of Riv (Fig 3A and 3B). The p-Akt levels in the presence of Riv were 1.4-fold higher than those observed in the absence of Riv at all time points examined (Fig 3B). The amount of p-ERK1/2 peaked at 10 min after the addition of NGF alone (Fig 3C and 3D). However, treatment with both NGF and Riv led to gradual increases in p-ERK1/2 of approximately 1.4- to 1.9-fold relative to that induced by NGF alone at all time points (Fig 3C and 3D). These results demonstrate that Riv upregulated the NGF-induced phosphorylation of Akt and ERK1/2 in PC12 cells.

### Effect of TrkA receptor antagonist on NGF-induced neurite outgrowth following enhancing effect of Riv

**Fig 4:**
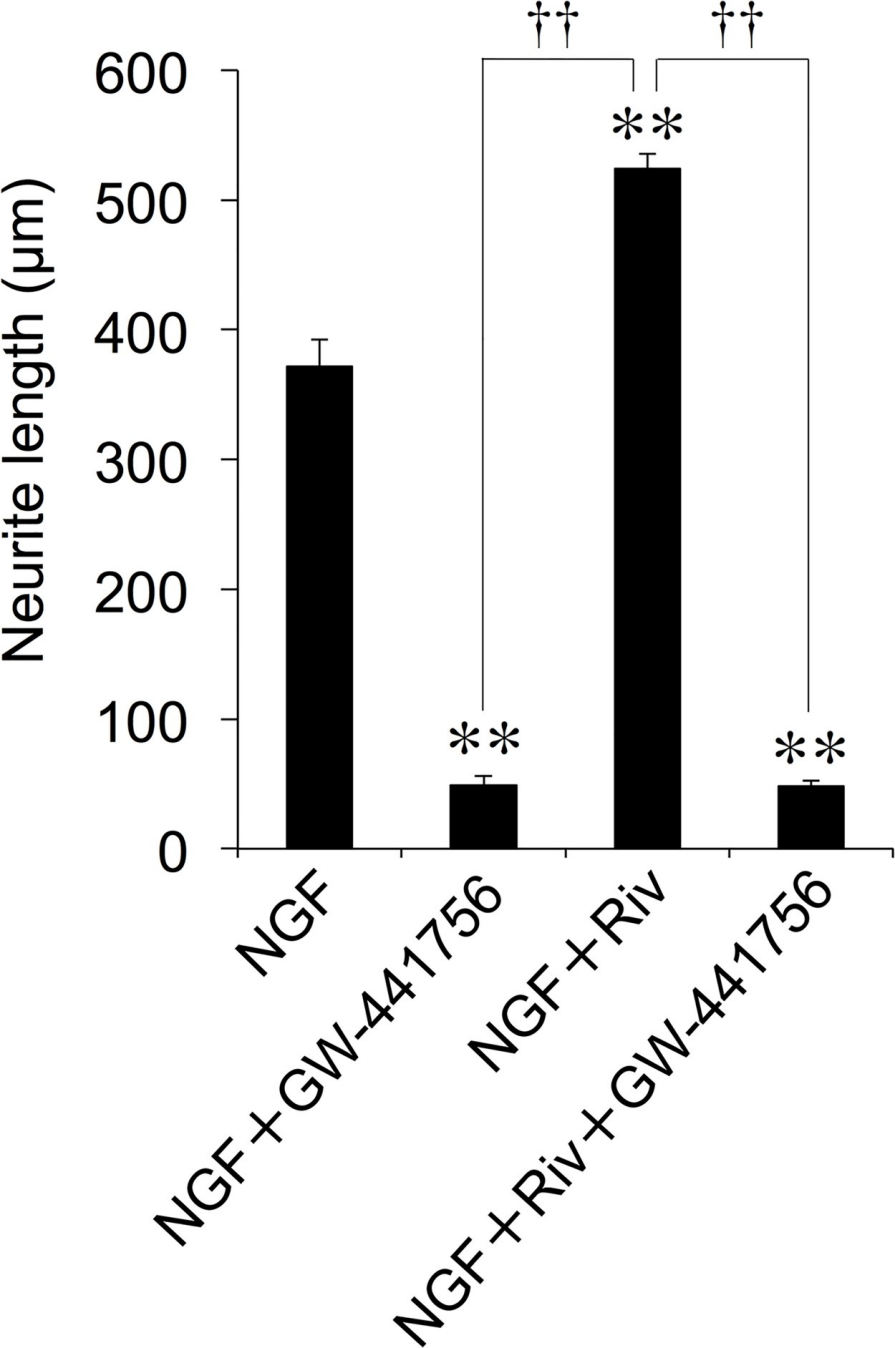
Effect of TrkA receptor antagonist on Riv-mediated enhancement of NGF-induced neurite outgrowth in PC12 cells. PC12 cells were pre-incubated in the presence or absence of GW-441756 (1 μM) for 4 h. NGF and Riv were added, and cells were incubated for an additional 24 h before neurite outgrowth assays. For each condition, values are reported as the mean ± SD for all PC12 cells included within five randomly chosen fields. **p < 0.01 compared with NGF, ^††^*p* < 0.01 compared with NGF+Riv).

TrkA is known as a high-affinity catalytic receptor for NGF, the binding of which mediates neurite outgrowth and cellular survival in neurons [36]. Hence, we investigated the effect of a selective TrkA antagonist, GW-441756, on Riv in NGF-induced neurite outgrowth. PC12 cells were treated with 1 μM GW-441756 for 4 h in the absence and presence of 100 μM Riv, after which they were incubated with NGF for a further 24 h. As shown in Fig 4, NGF induced neurite outgrowth in PC12 cells, and this was completely inhibited by GW-441756. Similarly, the Riv enhancement of NGF-induced neurite outgrowth was completely inhibited by treatment with GW-441756 (Fig 4). These findings suggest that Riv enhances NGF-induced neurite outgrowth via the TrkA receptor.

### Contribution of AChR to Riv-mediated enhancement of NGF-induced neurite outgrowth

**Fig 5:**
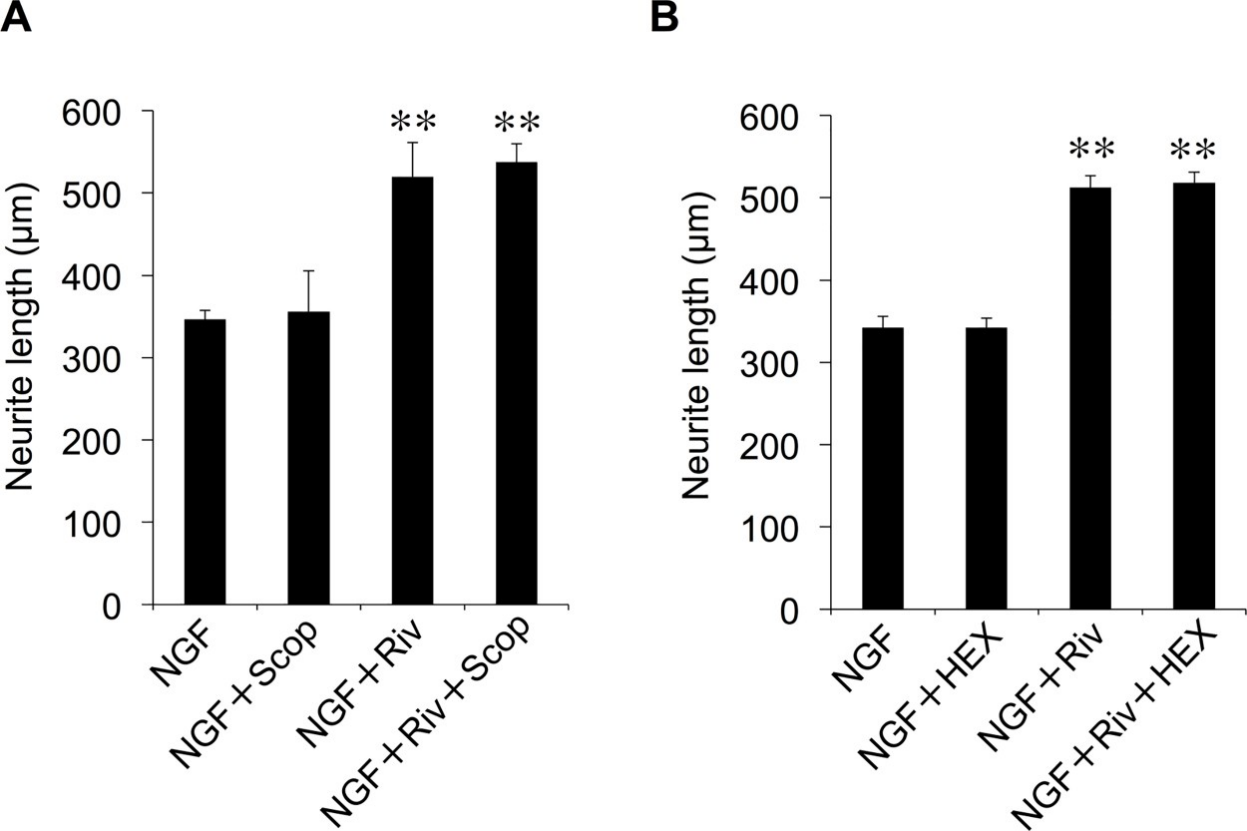
Effect of AChR antagonists on Riv-mediated enhancement of NGF-induced neurite outgrowth in PC12 cells. (**A**) PC12 cells were pre-incubated in the presence or absence of Scop (100 μM) for 4 h. (**B**) PC12 cells were pre-incubated in the presence or absence of HEX (100 μM) for 4 h. NGF and Riv were added, and cells were incubated for an additional 24 h before neurite outgrowth assays. For each condition, values are reported as the mean ± SD for all PC12 cells included within five randomly chosen fields. **p < 0.01 compared with NGF.

AChE inhibitors are known to have cholinergic system activity via AChR [9], and both nicotinic ACh receptors (nAChR) and muscarinic ACh receptors (mAChR) are expressed in PC12 cells [37,38]. We next investigated the effect of the AChR antagonists Scop and HEX on the Riv-mediated enhancement of NGF-induced neurite outgrowth. Scop is an mAChR antagonist that inhibits cholinergic transmission in the central nervous system [39], while HEX is an nAChR antagonist [40]. As shown in Fig 5A and 5B, neither Scop (100 μM) nor HEX (100 μM) inhibited NGF-induced neurite outgrowth. In addition, the enhancing effects of Riv on NGF-induced neurite outgrowth were not affected by treatment with Scop or HEX. These results suggest that AChR does not contribute to the enhancing effect of Riv on NGF-induced neurite outgrowth.

### Involvement of Sig-R in the effect of Riv

**Fig 6:**
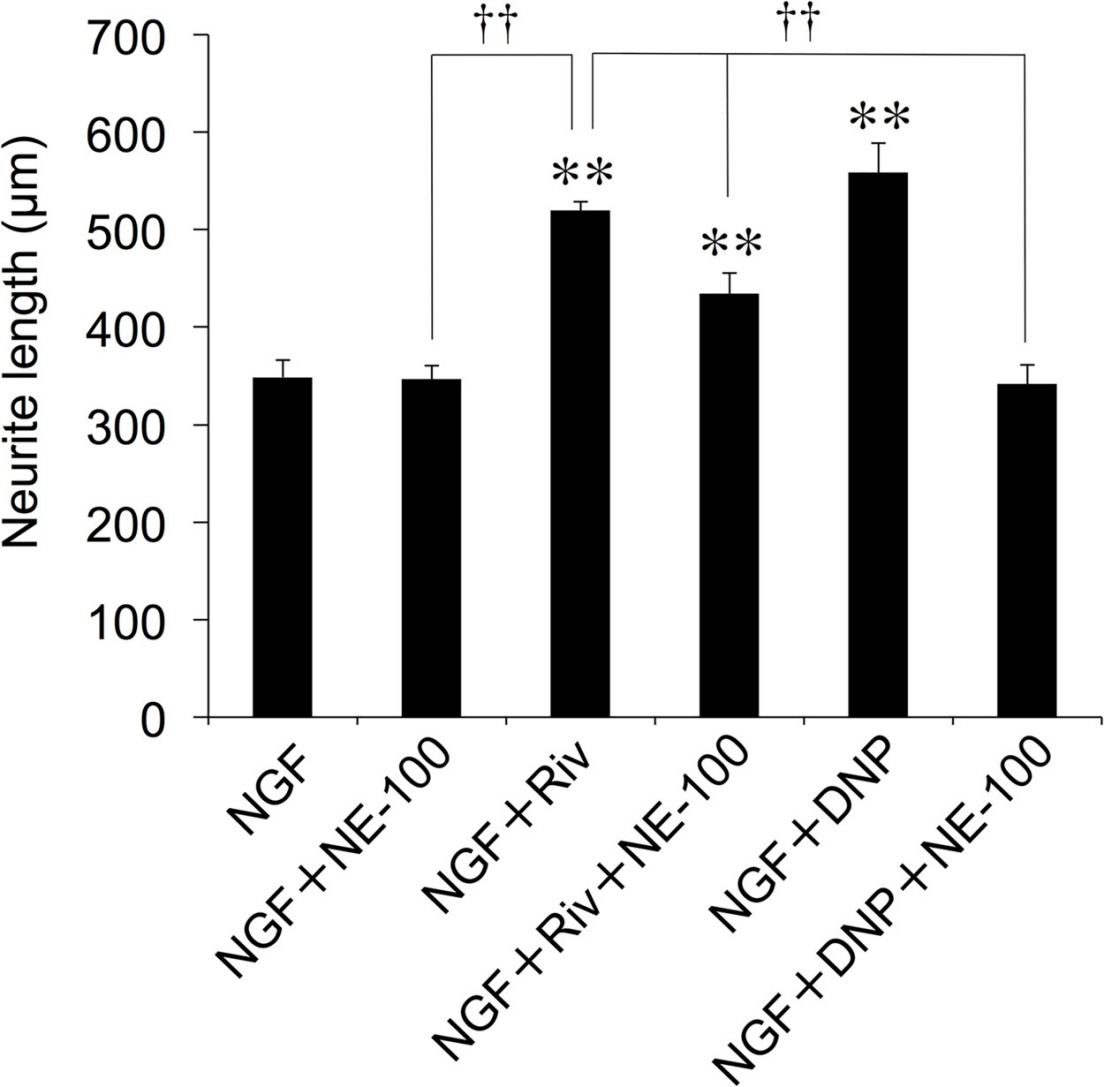
Effect of Sig-1R antagonist on Riv-mediated enhancement of NGF-induced neurite outgrowth in PC12 cells. PC12 cells were pre-incubated in the presence or absence of NE-100 (10 μM) for 4 h. NGF and Riv were added, and cells were incubated for an additional 24 h before neurite outgrowth assays. For each condition, values are reported as the mean ± SD for all PC12 cells included within five randomly chosen fields. **p < 0.01 compared with NGF, ^††^*p* < 0.01 compared with NGF+Riv.

The AD therapeutic agent DNP exerts its neuronal effects via high-affinity binding to Sig-1R [10]. In the present study, DNP (100 μM) also had an enhancing effect on NGF-induced neurite outgrowth (Fig 6). The effect of DNP was completely inhibited by treatment with NE-100, though NE-100 (10 μM) did not inhibit neurite outgrowth induced by NGF alone. We therefore examined whether the effect of Riv involves Sig-1R activity. The effect of Riv in enhancing NGF-induced neurite outgrowth was attenuated by treatment with NE-100, but this attenuation was weaker than that of DNP (49.7% decrease, Fig 6).

**Fig 7:**
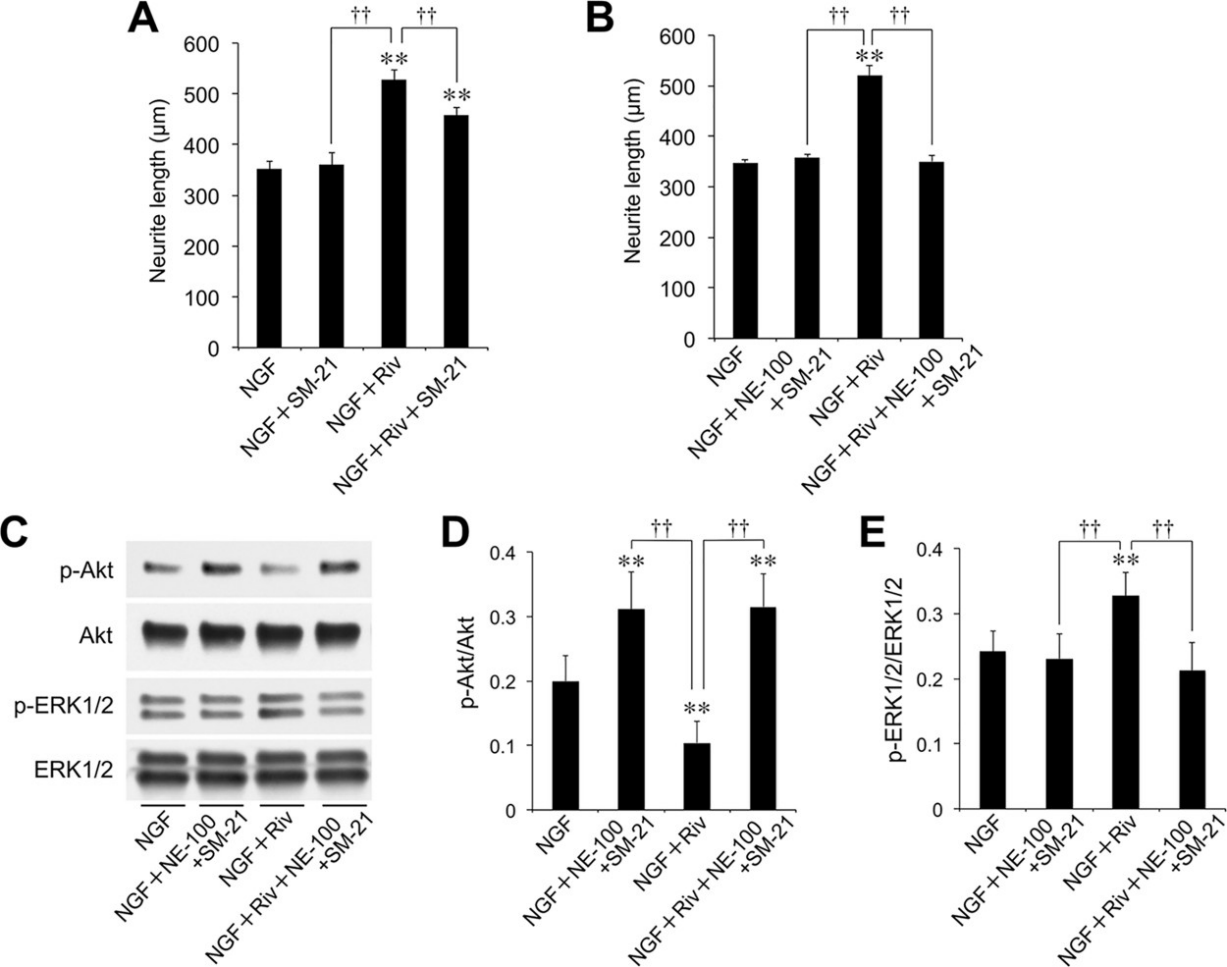
Effect of Sig-2R antagonist and additional Sig-1R antagonist on Riv-mediated enhancement of NGF-induced neurite outgrowth and phosphorylation of Akt and ERK1/2 in PC12 cells. (**A**) PC12 cells were pre-incubated in the presence or absence of SM-21 (10 μM) for 4 h. (**B, C**) PC12 cells were pre-incubated in the presence or absence of SM-21 (10 μM) and NE-100 (10 μM) for 4 h. NGF and Riv were added, and cells were incubated for an additional 24 h before neurite outgrowth and western blotting assays. (**C**) Total protein lysates were collected and subjected to western blotting for the assessment of the phosphorylation status of Akt and ERK1/2. (**D**, **E**) The intensity of the p-Akt polypeptide band was normalized to that of the total Akt band (p-Akt/Akt) (**D**), while the intensity of the p-ERK1/2 polypeptide band was normalized to that of the total ERK1/2 band (p-ERK1/2/ERK1/2) (**E**). For each condition, values are reported as the mean ± SD for all PC12 cells included within five randomly chosen fields. **p < 0.01 compared with NGF, ^††^*p* < 0.01 compared with NGF+Riv.

Next, we investigated the involvement of Sig-2R in the effect of Riv. SM-21 is known to be a selective antagonist of Sig-2R. SM-21 (10 μM) did not inhibit neurite outgrowth induced by NGF alone (Fig 7A). By contrast, the Riv-mediated enhancement of NGF-induced neurite outgrowth was partially inhibited by treatment with SM-21 (39.8% decrease, Fig 7A). Extending the above results, the enhancing effect of Riv on NGF-induced neurite outgrowth was completely inhibited following combined treatment with NE-100 and SM-21 (Fig 7B). These findings indicate that the cooperative enhancement of NGF-induced neurite outgrowth following treatment with Riv occurred via Sig-1R and Sig-2R. In addition, we evaluated the Riv-mediated enhancement of NGF-induced Akt and ERK1/2 phosphorylation in the presence and absence of Sig-1R and Sig-2R antagonists using western blotting. The phosphorylation of Akt by NGF was enhanced by NE-100 and SM-21 treatment in 24-h culture (Fig 7C and 7D), while the NGF-induced upregulation of p-Akt was attenuated upon the addition of Riv (Fig 7C and 7D). Moreover, the Riv-mediated change in NGF-induced Akt phosphorylation disappeared following treatment with a combination of NE-100 and SM-21 (Fig 7C and 7D). In contrast, Riv enhanced the NGF-induced phosphorylation of ERK1/2 (Fig 7C and 7E). However, Riv-enhanced phosphorylation of ERK1/2 was completely inhibited by treatment with the Sig-1R and Sig-2R antagonists (Fig 7C and 7E). These results suggest that Riv affects NGF-dependent Akt and ERK1/2 phosphorylation via Sig-1R and Sig-2R.

**Fig 8:**
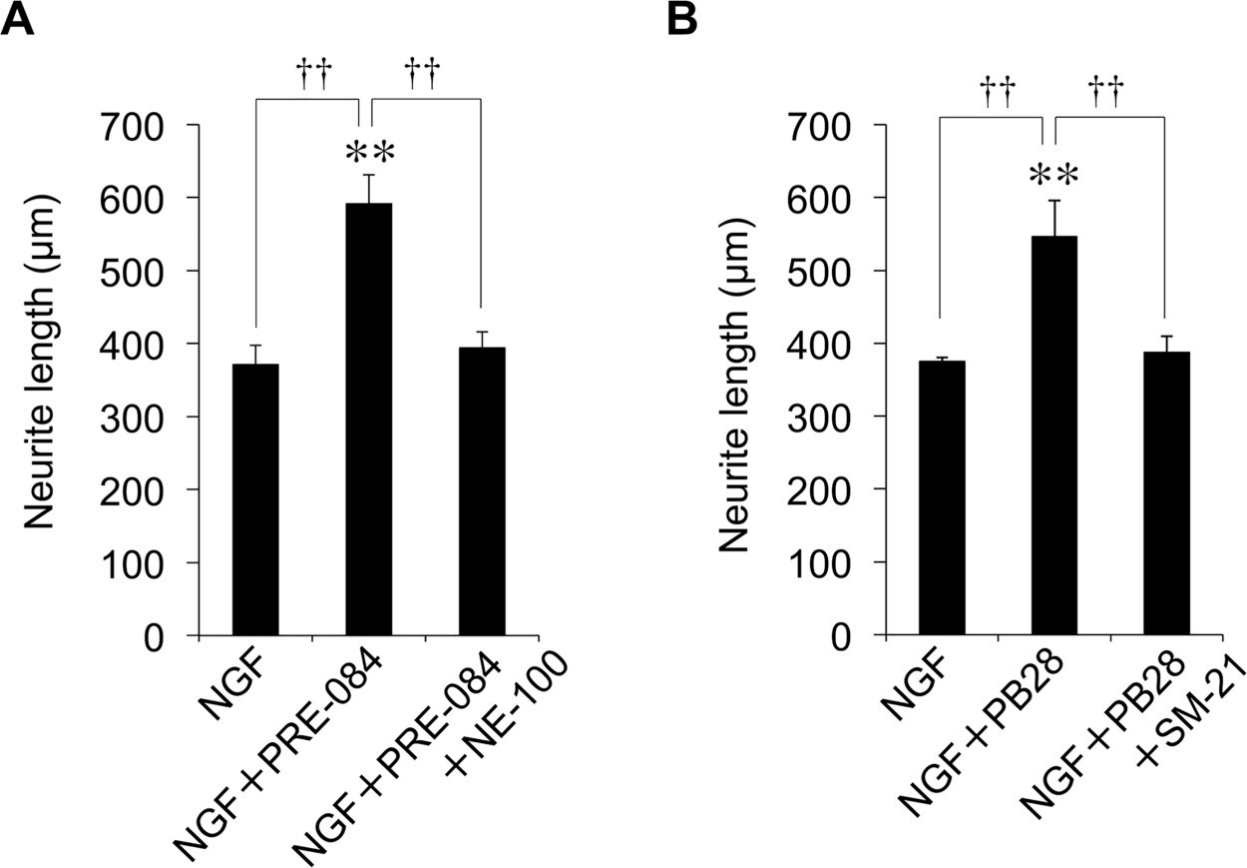
Effect of Sig-1R and Sig-2R agonist/antagonist on NGF-induced neurite outgrowth in PC12 cells. (**A**) PC12 cells were pre-incubated in the presence or absence of NE-100 (10 μM) for 4 h. NGF and PRE-084 (10 μM) were added, and cells were incubated for an additional 24 h before neurite outgrowth assays. (**B**) PC12 cells were pre-incubated in the presence or absence of SM-21 (10 μM) for 4 h. NGF and PB28 (10 μM) were added, and cells were incubated for an additional 24 h before neurite outgrowth assays. For each condition, values are reported as the mean ± SD for all PC12 cells within five randomly chosen fields **p < 0.01 compared with NGF, ^††^*p* < 0.01 compared with NGF+PRE-084 or NGF+PB28.

In addition, we evaluated the effect of Sig-1R and Sig-2R agonist on NGF-induced neurite outgrowth. PRE-084, a selective agonist of Sig-1R, enhanced NGF-induced neurite outgrowth at 10 μM (Fig 8A). This enhancement was completely inhibited by treatment with NE-100 (10 μM). The Sig-2R agonist PB28 also enhanced NGF-induced neurite outgrowth at 10 μM, and this enhancement disappeared upon treatment with SM-21 at 10 μM (Fig 8B). These results indicate that agonists of both Sig-1 R and Sig-2 R have potentiating effects on NGF-induced neurite outgrowth.

**Fig 9:**
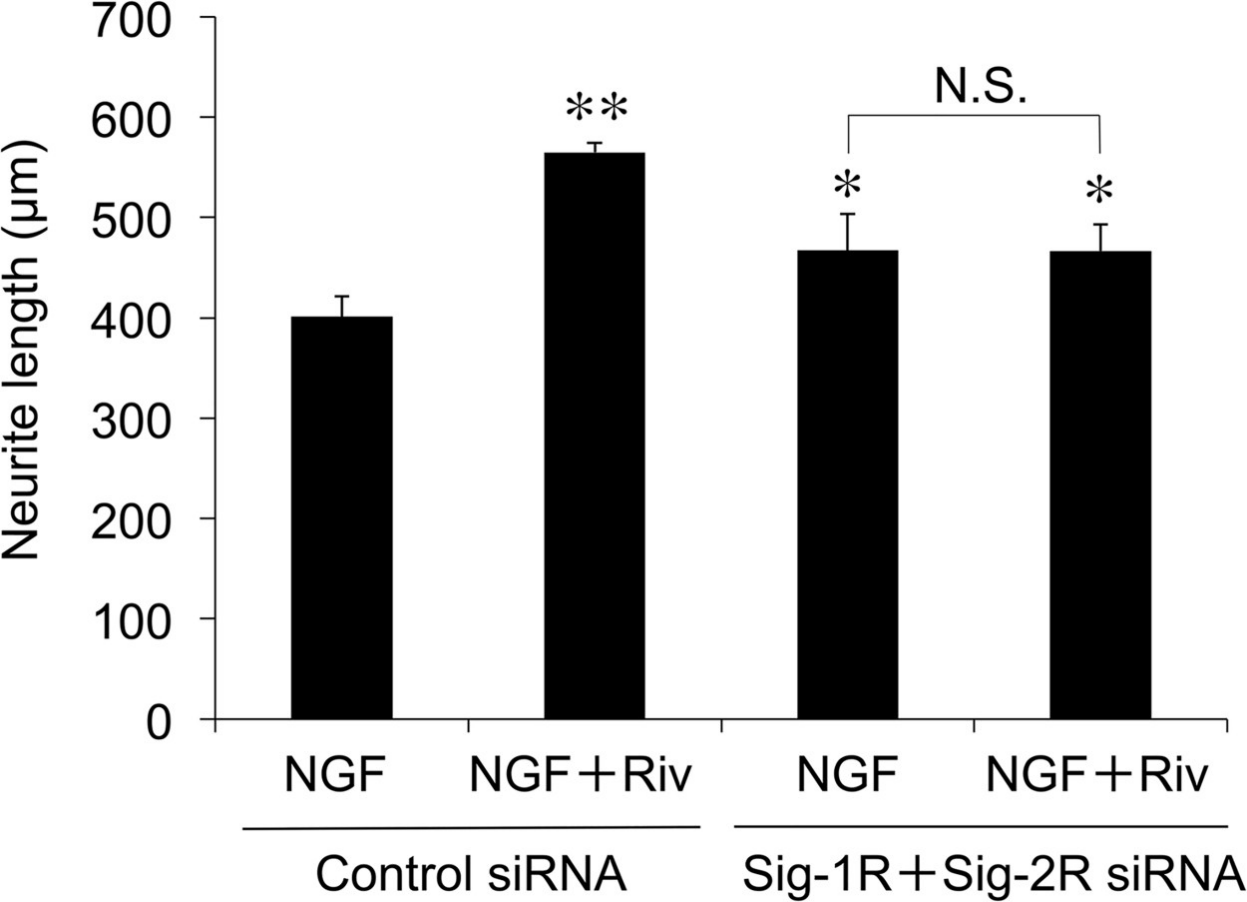
Influence of knockdown of Sig-1R and Sig-2R on Riv-mediated enhancement of neurite outgrowth in PC12 cells. PC12 cells were transfected with 10 nM siRNA (Sig-1R and Sig-2R siRNA). After 2 days of transfection, NGF and Riv were added, and cells were incubated for 24 h. For each condition, values are the mean ± SD for all PC12 cells within five randomly chosen fields. *p < 0.05; **p < 0.01 compared with control siRNA NGF. N.S., not significant.

To further confirm the participation of Sig-R in Riv-mediated enhancement of NGF-induced neurite outgrowth, we performed knockdown of Sig-1R and Sig-2R expression within PC12 cells. The knockdown of Sig-1R and Sig-2R was confirmed by quantitative RT-PCR (knockdown ratio >67%; data not shown). The enhancing effects of Riv on NGF-induced neurite outgrowth were not affected in control siRNA-transfected cells (Fig 9). In contrast, the Riv-mediated enhancement of NGF-induced neurite outgrowth was completely inhibited by the knockdown of both Sig-1R and Sig-2R within the cells. These findings strongly support the idea that Riv enhances NGF-dependent neurite outgrowth through Sig-R.

## Discussion

In this study, we demonstrated that Riv enhances NGF-induced neurite outgrowth in PC12 cells and that the effect of Riv was completely blocked by co-application of a Sig-1R or Sig-2R antagonist or siRNA. Riv alone does not cause neurite outgrowth in PC12 cells. However, Riv significantly enhances the E_max_ of neurite outgrowth following treatment with NGF, suggesting that Riv acts synergistically on NGF-induced neurite outgrowth in PC12 cells. This is the first report that Riv enhances NGF-induced neurite outgrowth through both Sig-1R and Sig-2R.

Several AChE inhibitors have been shown to have AchR-mediated effects [8,9]. Riv has also been reported to act on AChR [41]. However, the AChR antagonists Scop and HEX did not affect the Riv-mediated enhancement of NGF-induced neurite outgrowth. It is also reported that the mAChR agonist carbachol and nAChR agonist nicotine do not affect NGF-induced neurite outgrowth [42]. These results indicate that Riv enhances NGF-induced neurite outgrowth via an AChR-independent mechanism.

However, it is reported that some AChE inhibitors act on Sig-1R [43]. DNP exhibits affinity for Sig-1R and enhances NGF-induced neurite outgrowth in PC12 cells. In this study, the effects of DNP were completely inhibited by simultaneous addition of the selective Sig-1R antagonist NE-100. Phy, which does not have Sig-1R affinity, does not alter NGF-induced neurite outgrowth. By contrast, the enhancing effects of Riv on NGF-induced neurite outgrowth were attenuated by NE-100, although this effect was only partial, even at higher concentrations of NE-100 (data not shown). These results indicate that the Riv-mediated effect is partly associated with Sig-1R. It is well known that Sig-1R is an endoplasmic reticulum (ER) chaperone that exists at the mitochondria-associated ER membrane (MAM) [44]. Sig-1R binds to functional molecules such as inositol 1,4,5-trisphosphate receptor (IP3R), binding immunoglobulin protein (BiP) and Ras-related C3 botulinum toxin substrate 1 (Rac1)-GTPase, and shows chaperone activity [45]. Although BiP itself functions as a chaperone molecule, Sig-1R coassembles with BiP under normal physiological conditions [44]. Stimulation of Sig-1R by its agonists, such as cocaine and (+)-pentazocine, causes the dissociation of Sig-1R from BiP, independent of the effect of local calcium [44]. Sig-1R also shows chaperone activity against Rac1 and regulates neurite outgrowth and spine formation via Rac1-GTPase activity [46,47]. Therefore, it is speculated that activation of Sig-1R by Riv may trigger dissociation from BiP at the MAM. Furthermore, drugs with affinity for Sig-1R have been reported to have neuroprotective effects on depression and amyloid-β (Aβ)-induced neurotoxicity [31,48,49]. This finding shows that Riv may result in Sig-1R-mediated benefits in AD treatment.

Moreover, we investigated whether the effect of Riv involves Sig-2R. In this study, the Riv-mediated enhancement of NGF-induced neurite outgrowth was inhibited by SM-21, a Sig-2R antagonist. This suggests that the mechanism of Riv involves not only Sig-1R action but also Sig-2R action, unlike DNP. Sig-2R as a receptor remains poorly understood. However, the pharmacological function of Sig-2R has been gradually revealed by the identification of small molecules having an affinity for Sig-2R [25,50,51]. In addition, Alon et al. identified the gene that codes for Sig-2R as *TMEM97* [27]. Recently, it has been reported that specific ligands of Sig-2R inhibit Aβ oligomers binding to neurons [26]. Additionally, it was revealed that a selective Sig-2R agonist, DKR-1051, exerts neuroprotective effects by regulating Ca^2+^ levels in neurons [24]. Administration of DKR-1051 has also been reported to improve cognitive function in mice, suggesting that Sig-2R is a promising therapeutic target for AD [24]. In the present study, we have demonstrated that both the Sig-1R agonist PRE-084 and Sig-2R agonist PB28 have enhancing effects on NGF-induced neurite outgrowth. Therefore, it is highly possible that Riv-induced effects involve not only Sig-1R but also Sig-2R. Furthermore, we evaluated the effect of Riv upon the simultaneous addition of NE-100 and SM-21 to inhibit both Sig-1R and Sig-2R. Simultaneous addition of NE-100 and SM-21 completely abolished the Riv-mediated enhancement of NGF-induced neurite outgrowth and phosphorylation of Akt and ERK1/2. Moreover, the enhancing effects of Riv on NGF-induced neurite outgrowth were abolished in Sig-1R- and Sig-2R-knockdown PC12 cells. These results indicate that the effect of Riv is exerted via the cooperative action of Sig-1R and Sig-2R. However, the binding affinity of Riv to Sig-R has not yet been evaluated. While this study strongly suggests that Riv has an affinity for Sig-R, this has not been confirmed experimentally. Therefore, detailed binding affinity studies of Sig-R1 and/or Sig-R2 on Riv are warranted in the future.

The pharmacodynamic effects of Riv indicated that Riv altered the E_max_ (i.e., efficacy) but not the EC_50_ (i.e., potency) of NGF to induce neurite outgrowth. The fact that Riv did not change the EC_50_ of NGF suggests that the affinity of NGF for the TrkA receptor was not affected by Riv. In addition, GW-441756 treatment resulted in the complete inhibition of NGF-induced neurite outgrowth in both the presence and absence of Riv. During PC12 cell differentiation, Ras and PI3K signaling play key roles in the regulation of NGF-induced neurite outgrowth [35,52]. Akt and ERK1/2 are then activated downstream of Ras and PI3K, respectively [53,54]. In the present study, we observed a significant increase in neurite outgrowth following the addition of NGF, along with an increase in the phosphorylation/activation of Akt and ERK1/2. These results indicate that the enhancing effect of Riv on NGF-induced neurite outgrowth is dependent on the TrkA receptor pathway. Taken together, we speculate that Riv acts through Sig-1R and Sig-2R to increase the E_max_ of NGF-induced neurite outgrowth by increasing the number of NGF/TrkA receptor complexes and/or the activation of the downstream TrkA pathway to cellular responses. In AD therapy, there is a considerable amount of evidence to suggest that basal forebrain cholinergic neurons rely on NGF to maintain their survival, differentiation, connectivity, and function [4,55,56]. However, the decreased expression and immunoreactivity of the TrkA receptor has been observed in the basal forebrain cholinergic neurons of AD patients [57,58]. Thus, our findings indicate that the Riv-mediated enhancement of the NGF/TrkA receptor pathway may play an important role in AD therapy.

## Conclusions

The present study demonstrated that Riv potentiates NGF-induced neurite outgrowth in PC12 cells and that both Sig-1R and Sig-2R play a role in the mechanism of this effect. Therefore, it is thought that both Sig-1R and Sig-2R are involved in the pharmacological action of Riv in humans. Furthermore, agents such as Riv that target Sig-1R and Sig-2R may enhance the effect of NGF, playing an important role in the therapeutic treatment of AD in the future.
